# Detection of the novel HLA allele, HLA‐DRB1*08:112, identified in a Danish family

**DOI:** 10.1111/tan.14868

**Published:** 2022-11-07

**Authors:** Annette Plesner, Jacob Hald, Henrik Sengeløv, Helle Bruunsgaard

**Affiliations:** ^1^ Department of Clinical Immunology – 7631 Rigshospitalet, Copenhagen University Hospital Copenhagen N Denmark; ^2^ Department of Hematology, Bone Marrow Transplant Unit Rigshospitalet, Copenhagen University Hospital Copenhagen N Denmark; ^3^ Department of Clinical Medicine University of Copenhagen Copenhagen N Denmark; ^4^ Present address: Department of In Vivo Biology & Biomarkers Leo Pharma A/S Ballerup Denmark

**Keywords:** HLA‐DRB1*08:01, HLA‐DRB1*08:112, next‐generation sequencing, novel HLA‐allele

## Abstract

HLA‐DRB1*08:112 differs from HLA‐DRB1*08:01 in exon 2 at amino acid 62; asparagine to lysine substitution.

The HLA genes are highly polymorphic and play a key role in protective immunity, autoimmune diseases and in complications pertaining to transfusion medicine and transplantation inmmunology.^1^ In this report we present a novel HLA‐DRB1 allele, now named HLA‐DRB1*08:112, identified both in a Danish hematopoietic stem cell recipient and confirmed in family members as part of our routine immunological evaluation.

High‐resolution HLA typing of DNA was performed using the Illumina MiSeq next‐generation sequencing (NGS) platform. Genomic DNA was isolated from peripheral EDTA blood, using a Tecan Freedom EVO®‐HSM Workstation and ReliaPrep™ Large Volume HT gDNA isolation kit (A2751; Promega Corporation). In total 100 ng genomic DNA was used as input per reaction to amplify the HLA genes. Polymerase chain reaction, fragmentation and library preparation was performed using the NGSgo®‐AmpX HLAGeneSuite™ CE kit (7871662) with NGSgo‐AmpX Whole Gene for HLA‐DRB1 (7370622) and HLA‐DQB1 (7370512), NGSgo®‐LibrX Library Preparation Illumina (2342605) and NGSgo®‐IndX Indexed Adapters Illumina (4×24; 2342203 and 2342303). All laboratory protocols followed the manufacturer's instructions (GenDx) and library amplification and sequencing was performed using a Micro Flowcell (TG‐142‐1002) on the Miseq platform (Illumina, Inc.). The genotype of the sample was assigned using NGSengine version 2.18.0 from GenDx and based on the IPD‐IMGT/HLA Database version 3.40.0.^2^ Manual inspection of data was performed where data did not support automated assignment by NGS engine. At the base position giving rise to the novel gene the read depth was 5329 and the read distribution between C and G was 60% and 40%, respectively. To confirm the new type, informed consent was obtained from the patient to independently retype the same DNA sample and the same mutation was also confirmed when typing the DNA samples of family members.

A phased DNA contiguous sequence of 3580 bases was obtained, spanning all of exon 2 and 3, starting upstream in intron 1 (gDNA 5278) and continuing through to the first four bases of exon 4. Compared with HLA‐DRB1*08:01, the new allele had one nucleotide substitution in CDS position 273 (C– > G, codon 62) in exon 2 (Figure 1). This results in the nonconservative substitution of an asparagine (N) to a lysine (K). These two amino acids differ in their side chains given that asparagine has a neutral polar side chain typically forming hydrogen bonds, whereas lysine is positively charged at neutral pH and therefore has the propensity to form salt bridges. The substitution is located in the extracellular domain containing the peptide binding groove and could result in a mature protein with a conformational change compared with DRB1*08:01. It is beyond the scope of this report to decipher the potential functional effect of this substitution including allele localization and protein level.

**FIGURE 1:**
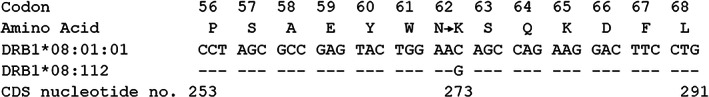
Sequence of the novel HLA‐DRB1*08:112 allele. Alignment of exon 2 nucleotide and amino acid sequences of HLA‐DRB1*08:01:01 and the novel HLA‐DRB1*08:112 allele. Dashes denotes identity to the DRB1*08:01:01 sequence. The new HLA‐DRB1 allele has 1 nucleotide substitution at CDS position 273 (C– > G, codon 62) in exon 2, resulting in an asparagine (N) to lysine (K) substitution.

The HLA genotype was resolved to be HLA‐A*01:01:01, *24:02:01*; B*08:01:01, *39:06:02*; C*07:01:01, *07:02:01*; DRB1*03:01:01, *08:112*; DRB3*01:01:02; DQA1*04:01:01, *05:01:01*; DQB1*02:01:01, *04:02:01*; DPA1*01:03:01; DPB1*04:01:01.

The sequence of the new HLA‐DRB1*08:112 allele has been submitted to the IPD‐IMGT/HLA Database and GenBank and assigned the accession numbers HWS10060078 and OL770265, respectively.

The name DRB1*08:112 has been officially assigned by the WHO Nomenclature Committee for Factors of the HLA System in February 2022. This follows the agreed policy that, subject to the conditions stated in the most recent Nomenclature Report, names would be assigned to new sequences as they are identified.^3^ Lists of new names will be published in the following WHO Nomenclature Report.

## AUTHOR CONTRIBUTIONS

Annette Plesner: Performed laboratory analyses; submitted the new sequence to IMGT/HLA database and Genbank; responsible for communication with the WHO nomenclature committee; writing and submission of the paper. Jacob Hald: Performed laboratory analyses; submitted the new sequence to IMGT/HLA database and Genbank; participated in writing the paper. Henrik Sengeløv: JACIE Clinical Program Director; responsible for patient treatment. Helle Bruunsgaard: EFI director with medical responsibility for HLA analyses and medical director of donor registry; participated in writing the paper.

## CONFLICT OF INTEREST

The authors declare no conflict of interest.

## Data Availability

The data that support the findings of this study are openly available in GenBank at https://www.ncbi.nlm.nih.gov/genbank/, reference number OL770265.
